# Associations Between Lipid Traits and Breast Cancer Risk: A Mendelian Randomization Study in African Women

**DOI:** 10.1002/cam4.70928

**Published:** 2025-05-02

**Authors:** Emmanuel Owusu Ansah, Foster Kyei, Caleb Frimpong Opoku, Andrews Danquah, Kwadwo Fosu, Emmanuel Boateng Agyenim, Daniel Sakyi Agyirifo

**Affiliations:** ^1^ Department of Molecular Biology and Biotechnology University of Cape Coast Cape Coast Ghana; ^2^ Gene Therapy Programme Thrivus Institute for Biomedical Science and Technology Cape Coast Ghana; ^3^ School of Medical Sciences University of Cape Coast Cape Coast Ghana; ^4^ Dormaa Presbyterian Hospital Dormaa Ahenkro Ghana; ^5^ West African Center for Cell Biology of Infectious Pathogens University of Ghana Legon Ghana; ^6^ Department of Biochemistry, Cell and Molecular Biology University of Ghana Legon Ghana; ^7^ Department of Virology Noguchi Memorial Institute for Medical Research Accra Ghana

**Keywords:** breast cancer, genetics, instrumental variables, lipids, Mendelian randomization

## Abstract

**Background:**

Blood lipids are implicated in the development of breast cancer (BC), though the genetic connection remains unclear, particularly in African populations. Observational studies on this topic are limited by confounding factors and reverse causation, potentially affecting the reliability of findings.

**Methods:**

We applied univariate and multivariable two‐sample Mendelian randomization to assess the causal association between blood lipids (total cholesterol [TC], high‐density lipoprotein [HDL], low‐density lipoprotein [LDL], and triglycerides [TG]) and BC. Summary‐level data for lipid traits were sourced from the Africa Wits‐INDEPTH partnership for Genomic Research (AWI‐Gen) (*N* = 10,603 women). BC data were obtained from the largest genome‐wide association study of BC in African women, comprising 18,034 bc cases and 22,104 controls.

**Results:**

Our analysis revealed that genetically predicted TG was associated with a decreased BC risk (OR = 0.73, 95% CI = 0.56–0.95, *p* = 0.018. In contrast, no significant associations were found between TC, HDL, or LDL levels and BC risk: TC (OR = 1.04, 95% CI = 0.93–1.18, *p* = 0.467), HDL (OR = 1.29, 95% CI = 0.93–1.79, *p* = 0.121), and LDL (OR = 1.04, 95% CI = 0.90–1.20, *p* = 0.577). After adjusting for the effects of other lipid traits, the association between TG and BC was attenuated, and no associations were observed for TC, HDL, or LDL. No causal relationship was found between lipid traits and BC subtypes.

**Conclusions:**

This study provides evidence that elevated triglycerides may be associated with a reduced risk of BC, whereas no significant associations were observed for TC, HDL, or LDL. Further research is needed to better understand the underlying mechanisms and potential clinical implications of these findings.

## 1 Introduction

Breast cancer (BC) is the second leading cause of cancer‐related deaths among women worldwide, making it essential to understand its risk factors for effective prevention and treatment [1]. Over the past two decades, BC incidence has risen by approximately 0.33% per year, increasing from 876,990 to 2,002,350 cases globally [2]. In 2022, there were 2.3 million new cases and over 666,000 deaths worldwide, with sub‐Saharan Africa contributing 146,130 cases and 71,662 deaths [3]. Notably, Africa now faces the highest age‐standardized BC incidence globally, with sub‐Saharan regions experiencing the highest burden [4]. According to the World Health Organization (WHO), the incidence and mortality rates in sub‐Saharan Africa continue to rise, with projected increases of 85.7% in new cases and 89% in deaths by 2040 [5]. Given this trajectory, integrated efforts focusing on risk reduction, early detection, and improved treatment options are critical for controlling BC and reducing its impact on women's health.

BC is a heterogeneous disease classified into distinct subtypes based on the immunohistochemical expression of hormone receptors. These subtypes include estrogen receptor‐positive (ER+), progesterone receptor‐positive (PR+), human epidermal growth factor receptor 2‐positive (HER2+), and triple‐negative breast cancer (TNBC), which lacks all three receptors [6]. These subtypes differ in gene expression patterns, influencing tumor aggressiveness, treatment response, and prognosis [7, 8]. TNBC, the most aggressive subtype, is more prevalent among women of African ancestry [9]. The heterogeneity presents a significant challenge in understanding their pathological variations and behaviors. As a result, analyzing key factors that influence risk, such as lipid metabolism, holds promise for identifying novel biomarkers, refining risk prediction models, and developing more effective prevention and treatment strategies.

Lipid metabolism plays a crucial role in cancer progression, as cancer cells exploit lipids for energy production, proliferation, and metastasis [10]. In particular, triglycerides (TG) serve as a primary source of fatty acid oxidation, a metabolic pathway that enhances cancer cell survival and therapy resistance [11]. However, the relationship between TG levels and BC risk remains inconsistent across populations and BC subtypes. Some studies report a positive association between TG levels and BC [12], whereas others suggest a potential protective effect [13, 14]. For example, a prospective cohort study by Furberg et al. [15] found that elevated TG levels were linked to an increased risk of postmenopausal BC in Norwegian women. In contrast, genetic studies have suggested that elevated TG levels might have a protective effect against BC, although results vary depending on BC subtypes and population characteristics [16].

In addition to TG metabolism, cholesterol metabolism, which includes the regulation of low‐density lipoprotein (LDL), high‐density lipoprotein (HDL), and total cholesterol (TC), plays a critical role in BC pathogenesis. LDL, a major component of TC, has been shown to promote cancer cell proliferation and migration by influencing the tumor microenvironment and hormone signaling [17]. In contrast, HDL is believed to exert protective effects because of its anti‐inflammatory and antioxidant roles [18]. However, the relationship between these lipid profiles and BC risk remains complex. Some studies report a positive association between LDL and BC risk [19], whereas others find no significant relationship [20]. Similarly, although higher HDL has been inversely associated with BC risk [21], evidence remains inconsistent. The role of lipid metabolism, including TC, HDL, LDL, and TG, in BC development is still debated, with conflicting findings complicating definitive conclusions.

Given these inconsistencies, population‐specific research is particularly critical in sub‐Saharan Africa, where BC cases are often diagnosed at advanced stages, leading to poorer outcomes [22]. Although obesity is a recognized BC risk factor in African populations, the role of lipid dysregulation remains underexplored. Few observational studies have examined the associations between lipid traits and BC risk in African populations. For example, Akinyemiju et al. found that elevated TG levels could increase overall BC risk, whereas low HDL was associated with TNBC and high LDL with ER‐negative BC [23]. A retrospective case–control study also showed that high‐LDL and low‐HDL levels were associated with increased risk of ER− and TNBC, respectively, whereas elevated TG and TC were linked to higher BC risk, particularly among postmenopausal women [23]. Similarly, a study in Ghana observed that BC patients had significantly higher TC, TG, and LDL cholesterol but lower HDL cholesterol compared to healthy controls [24].

However, observational studies are prone to confounding, making it difficult to determine causal relationships. Factors such as smoking, genetic predisposition, alcohol consumption, obesity, and socioeconomic status may obscure these associations [25, 26, 27]. Additionally, residual confounding from clinical and demographic variables—such as age, menopausal status, breast density, and tumor histology—could further complicate interpretation [13]. On the other hand, genetic studies, which can help address confounding and establish causality, have predominantly been conducted in European and Asian populations. Given genetic and environmental differences that influence lipid metabolism, findings from these populations may not apply to African populations. To address these limitations, robust causal inference methods are needed. To our knowledge, no study has systematically examined the potential genetic causation between serum lipid profiles and BC in African populations.

Mendelian randomization (MR) is a widely used technique to determine whether specific genetic variants have a causal effect on a particular trait or disease. MR leverages genetic variants randomly assigned at conception as instrumental variables (IV) [28]. By analyzing these genetic variants about an exposure and its outcome using genome‐wide association study (GWAS) data, MR can identify whether the exposure causes the outcome. This approach minimizes the influence of confounders and biases, providing a clearer insight into causal relationships [28].

This study aims to determine the causal relationship between lipid traits (LDL, HDL, TC, and TG) and BC susceptibility in African women using a two‐sample bidirectional MR approach. By leveraging the largest GWAS dataset available for BC in African women (18,034 cases and 22,104 controls) [29], we assessed whether lipid levels influence overall BC risk and its subtypes. Blood lipids are important biomarkers in cancer metabolism, and our findings may provide insights into lipid‐related mechanisms in BC, potentially informing targeted prevention strategies.

## 2 Methods

### 2.1 Overview of the Study Design

**FIGURE 1:**
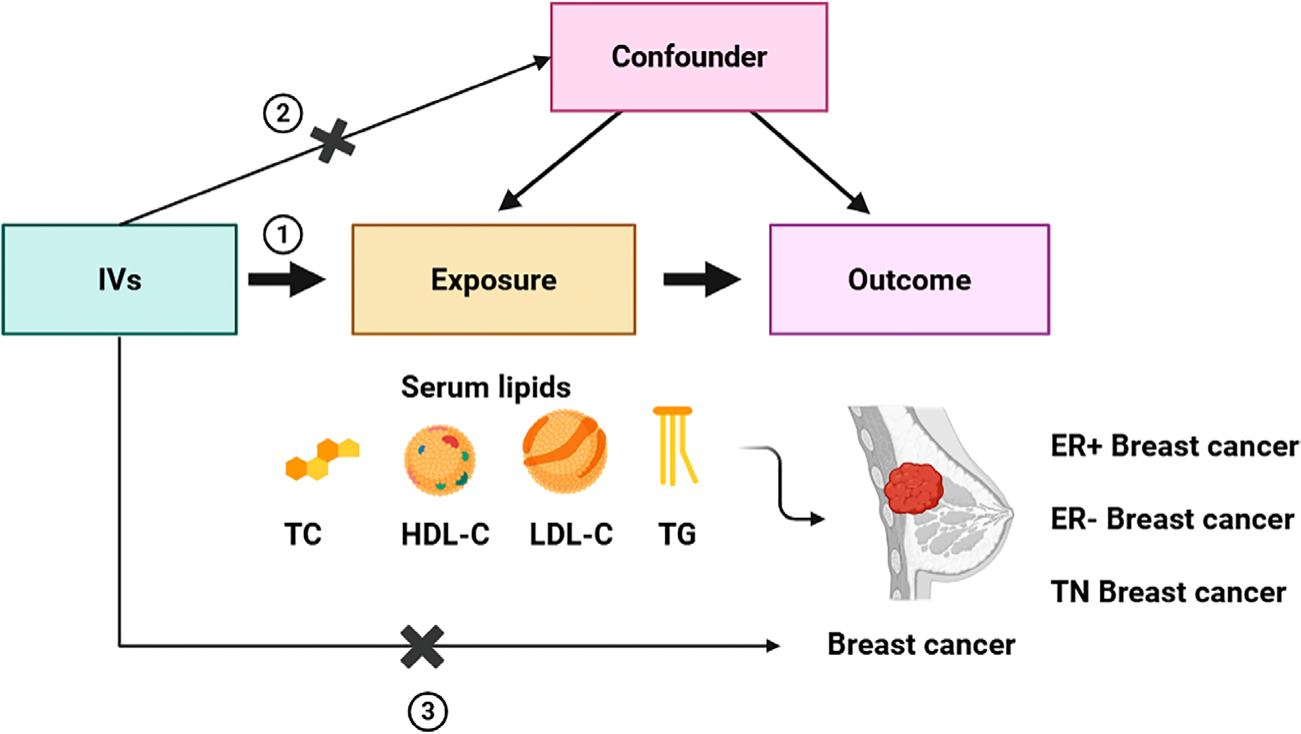
Study overview. MR depends on three key assumptions. The exposures in the study were serum lipids, whereas the outcomes were breast cancer and its subtypes. HDL‐C, high‐density lipoprotein cholesterol; LDL‐C, low‐density lipoprotein cholesterol; TC, total cholesterol; TG, total triglyceride.

The main analysis focused on the causal relationship of the lipid level with BC and its molecular subtypes. Specifically, we investigated the causal effect of various blood lipids (TC, HDL, LDL, and TG), individually, on the development of BC. These included 16 MR analyses using summary‐level data from large‐scale meta‐analyses of GWAS. In addition, a reverse analysis was performed to understand whether BC has a possible causal influence on lipids. Subsequently, a multivariate analysis was performed to determine the independent effect of each lipid type on BC. The three main assumptions underlying MR, as illustrated in Figure 1, are: first, IVs must be strongly associated with the exposure being studied; second, these IVs must not be linked to any confounding factors that could influence the relationship between the exposure and outcome; and third, genetic instruments should influence the outcome only through exposure. Details of sources of data used in this manuscript are summarized in Table S1. To reduce population stratification, all analyses were restricted to the African population only.

### 2.2 GWAS Data Sources

IVs for this study were derived from large‐scale GWAS conducted on individuals of African ancestry. Genetic instruments for lipid traits were obtained from two key studies: the African Partnership for Chronic Disease Research (APCDR) and the Africa Wits‐IN‐DEPTH Partnership for Genomics Studies, which included up to 24,215 participants. For BC outcomes, we used a recent GWAS dataset comprising 18,034 cases and 22,104 controls. The outcomes examined included general BC (18,034), ER+ (*n* = 9304), ER− (*n* = 4924), and TNBC (*n* = 2860). These outcome variables were defined by the African Ancestry Breast Cancer Genetic (AABCG) Consortium. Genotyping in these studies was performed using Illumina arrays or the Multiethnic Genotyping Array (MEGA). Rigorous quality control (QC) procedures were applied to both datasets, including the removal of single nucleotide polymorphisms (SNPs) with missingness > 0.05, minor allele frequency (MAF) < 0.01, and Hardy–Weinberg equilibrium (HWE) *p* value < 0.0001. Additional steps included imputation to the 1000 Genomes Project reference panel and adjustments for age, study design, and the first five principal genetic components. Ethical approval and participant consent were obtained in the original studies. All lipid datasets are available for download from the IEU OpenGWAS database (https://gwas.mrcieu.ac.uk/datasets/), and BC datasets can be accessed from https://www.ebi.ac.uk/gwas/studies. Additional information on GWAS can be found in the original studies [29, 30].

### 2.3 Extraction of SNPs Associated With Lipid Traits

We identified SNPs associated with each lipid trait from MRCIEU at a genome‐wide significance threshold (*p* < 5 × 10^−8^). To ensure the independence of IVs, SNPs in the disequilibrium of the linkage (LD) with each other were removed using an LD pruning threshold of *r* ^2^ = 0.001 and a kilobase (kb) threshold of 1000. The SNPs in the lipid datasets underwent screening, and consistency was ensured by harmonizing the direction of effect values between the exposure and outcome data. Ambiguous SNPs with incompatible alleles (e.g., A/G vs. A/C) were excluded from the analysis. Palindromic SNPs with intermediate allele frequencies (between 0.45 and 0.55) were also removed to minimize potential confounding effects that could violate the second MR assumption [31]. To assess the strength of selected SNPs, we calculated the *F*‐statistic (*F* = beta^2^/SE^2^) for each instrumental variable. IVs with an *F*‐statistic below 10 were considered weak instruments and therefore excluded from the analysis [32].

### 2.4 Quality Control and Data Standardization

Quality control (QC) of the BC summary statistics was carried out following the guidelines outlined by Murphy et al. [33]. We utilized MungeSumstats, a Bioconductor R package, to standardize and process the summary statistics. MungeSumstats uses automated QC procedures to ensure data consistency and accuracy. First, we standardized the column headers and checked the consistency of the dataset, confirming that the alleles were correctly represented and aligned with the reference genome. Nonbiallelic SNPs were removed, and any missing SNP IDs were imputed based on base pair positions and chromosome numbers. Identified indels, as well as duplicated RSIDs and base pair positions, were excluded from the dataset. Next, we verified that the directionality of the effect alleles matched the reference genome. Any discrepancies in allele alignment were corrected by flipping the effect columns as needed. We performed a SNP liftover from hg38 to hg19 to ensure that the summary statistics were correctly aligned with the genomic coordinates used in our exposure dataset. All genetic data were based on the 19th edition of the human genome reference sequence (HG19/GRCh37). Additionally, we renamed columns to accurately reflect allele frequencies and converted the summary statistics into GenomicRanges format, facilitating seamless integration with downstream genomic analyses.

### 2.5 Univariable Mendelian Randomization Analysis

We conducted two‐sample MR analyses using inverse variance weighted (IVW), weighted median (WM), and MR‐Egger models to estimate the causal relationship between lipid traits (TG, TC, HDL, and LDL) and the risk of BC [34, 35, 36]. We used the random effect IVW method as the main effect size estimator [37]. The IVW regression model combines genetic variant‐specific causal estimates weighted by the inverse of their variances [37]. The approach assumes the validity of the genetic instruments under the assumptions of no heterogeneity and no horizontal pleiotropy and, therefore, provides robust estimates of the causal effects. In the case of heterogeneity but no horizontal pleiotropy, the weighted median method was applied [38]. This method provides reliable estimates of the causal effect if at least half the weight comes from valid variants. However, when heterogeneity, or variation in causal estimates across genetic variants, and horizontal pleiotropy, where genetic variants influence multiple traits, were detected in our MR analyses, we performed the MR‐Egger regression method for our analysis. Specifically, MR‐Egger regression allows for an intercept term, which estimates the average pleiotropic effect across all variants used as instruments, to address biases induced by pleiotropy. Other MR algorithms, such as those for the simple median and simple mode described by Bowden [36], were also applied in this study to further assess the robustness of these findings.

### 2.6 Multivariable Mendelian Randomization Analysis

Since lipid traits are genetically related, we used multivariable Mendelian randomization (MVMR) to assess the direct effects of lipid traits on BC outcomes, following the method described by Sanderson et al. [28]. Here, we retrieved genetic‐associated variants for all the exposures across their summary datasets. The SNPs for all lipid traits were combined and we then filtered for genome‐wide significance (*p* < 5 × 10^−8^) and for linkage disequilibrium (*r* ^2^ < 0.001). To test for the presence of weak instruments, we evaluated the strength and validity of IVs. We also performed horizontal pleiotropy testing using conventional *Q*‐statistic estimation. Information on SNPs for the exposure and outcome is listed in Table S1.

### 2.7 Sensitivity Analyses

Heterogeneity among genetic variants was assessed using Cochrane's *Q* value of the IVW method, with *p* < 0.05 indicating significant heterogeneity [39]. We used the MR‐Egger intercept to check whether horizontal pleiotropy influenced our results [40]. A *p* value > 0.05 suggested that pleiotropy was not a significant factor. Furthermore, the Mendelian Randomization Pleiotropy RESidual Sum and Outlier (MR‐PRESSO) method was used to detect and correct outliers in the analysis [41]. The MR‐PRESSO approach is derived from the IVW method but includes the removal of genetic variants whose specific causal estimates deviate from those of other variants [41]. Nonetheless, a leave‐one‐out approach (LOO) was employed to evaluate the effect of each exposure SNP on the outcome of the MR analysis [42]. We achieved this by removing variants one by one from the analysis and re‐estimating the causal effect. The results were presented as odds ratios (OR) and 95% confidence intervals (CI), providing an estimate of how lipid traits influence the probability of developing BC [43]. A *p* value < 0.05 suggests that the observed association between lipid traits and the likelihood of developing BC is unlikely to be due to chance alone. All statistical analyses were performed in the R software version 4.2.2, using packages including TwoSampleMR, MVMR, MR‐PRESSO, and MendelianRandomization [44] (Table 1).

**TABLE 1:** Characteristics of the exposure variables.

GWAS ID	Trait	Cases	Controls	SNPs (number)	Population	Year	PMID
GCST90101747	Total cholesterol	24,612	—	21,307,140	African	2022	35546142
GCST90101746	High‐density lipoprotein	24,616	—	21,361,416	African	2022	35546142
GCST90101745	Low‐density lipoprotein	24,515	—	21,372,748	African	2022	35546142
GCST90101748	Triglyceride	24,600	—	21,308,171	African	2022	35546142
GCST90296719	All BC outcome	18,034	22,104	15,344,198	African	2024	38741014
GCST90296720	ER+ outcome	9304	19,024	15,369,976	African	2024	38741014
GCST90296721	ER− BC outcome	4924	17,058	15,333,374	African	2024	38741014
GCST90296722	TNBC outcome	2860	16,262	15,320,899	African	2024	38741014

## 3 Results

### 3.1 Single‐Trait MR in BC

In our study, we investigated the relationship between four lipid traits (i.e., TC, HDL, LDL, and TGs) and BC risk using data from the AWI‐GEN study. In the forward analysis, we identified 16 SNPs, 8 SNPs, 14 SNPs, and 6 SNPs associated with TC, HDL, LDL, and TG, respectively, at a genome‐wide significance level (*p* < 5 × 10^−8^) (Table S1). In reverse analysis, six SNPs related to BC were selected as IVs. *F*‐statistics for all selected traits exceeded 10, indicating no evidence of weak instrument bias. These SNPs explained approximately 0.2%, 0.4%, 3.6%, and 3.7% of TG, HDL, TC, and LDL variance, respectively (Table S1).

**FIGURE 2:**
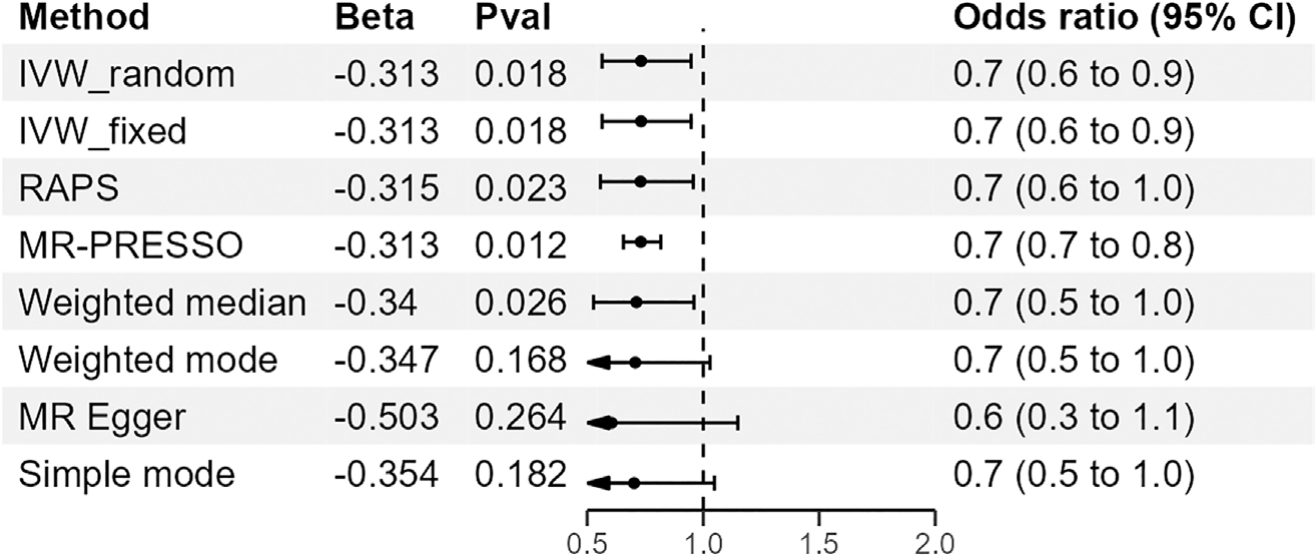
Estimates of TG levels on the risk of BC. CI, confidence interval; IVW_fixed, fixed effects inverse‐variance weighted; IVW_random, random effect inverse‐variance weighted; MR‐PRESSO, MR‐pleiotropy residual sum and outlier; Pval, *p* value; RAPS, robust adjusted profile score.

**FIGURE 3:**
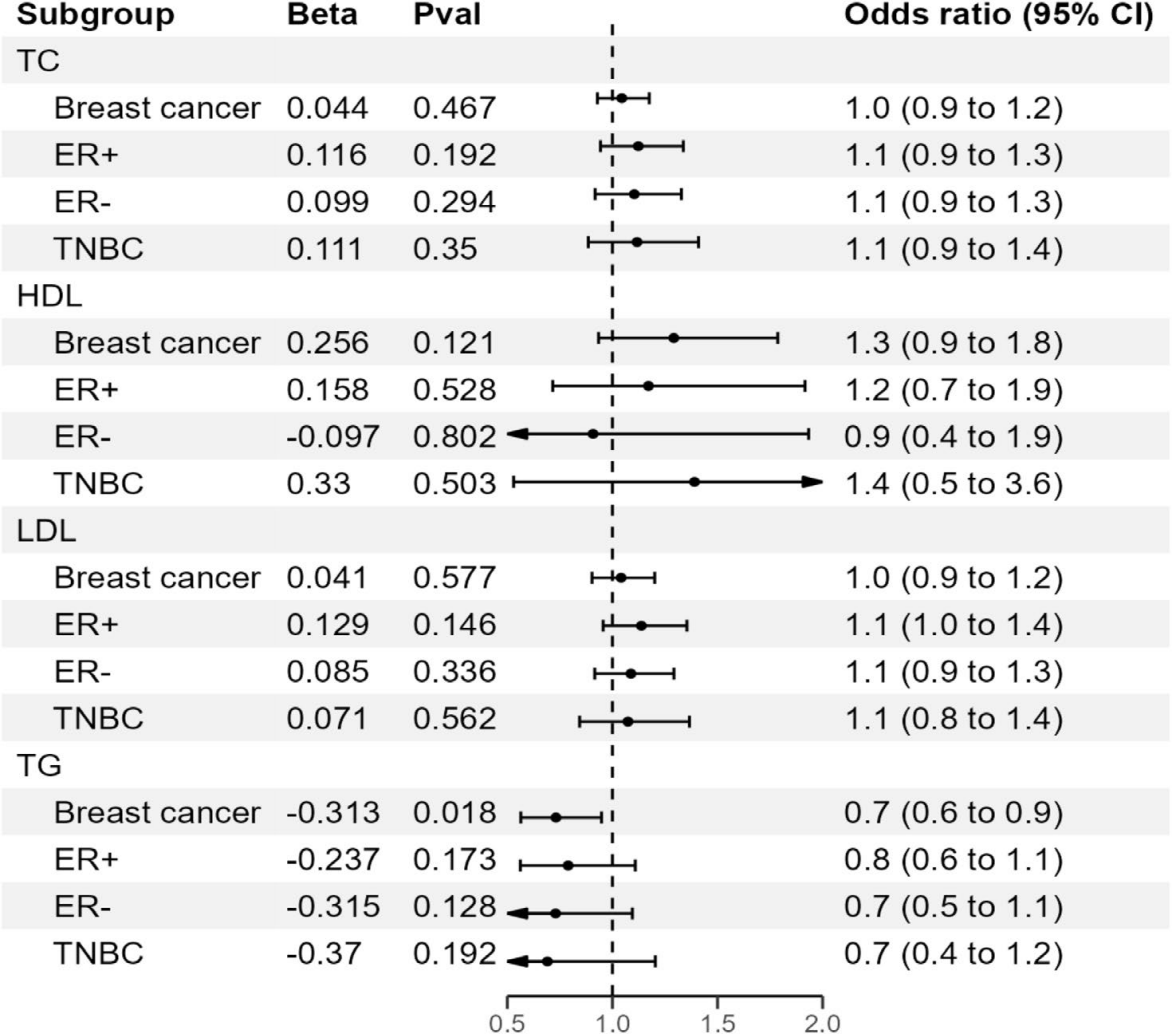
Estimates of triglyceride levels on the risk of breast cancer and its subtypes. CI, confidence interval; ER−, estrogen negative; ER+, estrogen positive; HDL, high‐density lipoprotein; LDL, low‐density lipoprotein; TC, total cholesterol; TG, triglycerides; TNBC, triple‐negative breast cancer.

In univariable MR analyses, TG was found to be a protective factor against BC. The IVW method indicated that a 1‐standard‐deviation increase in TG was associated with an approximately 0.7‐fold decrease in the risk of overall BC (OR = 0.73, 95% CI = 0.56–0.95, *p* = 0.018; Figure 2, Table 2). To further confirm our results, we conducted additional MR analyses using various methods, including MR‐PRESSO, IVW fixed effect method, MR‐Egger, weighted median, and robust adjusted profile scores (RAPs) methods. The negative association was consistent across all these methods (Figure 3 and Figure S2).

**TABLE 2:** MR results for the relationship between lipid traits and breast cancers. The MR analysis was performed through the TwoSampleMR packages (version 0.6.6) in R (version 4.2.2). All statistical tests were two sided. *p* < 0.05 was considered significant.

Risk factors	Overall BC	ER+ BC	ER− BC	TNBC
	OR (95% CI)	OR (95% CI)	OR (95% CI)	OR (95% CI)
**Total cholesterol (mg/dL)**				
Inverse variance weighted	1.04 (0.93–1.18)	1.12 (0.94–1.34)	1.10 (0.92–1.33)	1.12 (0.89–1.41)
Weighted median	1.06 (0.91–1.23)	1.14 (0.93–1.39)	1.14 (0.89–1.45)	1.17 (0.87–1.58)
MR‐Egger	1.16 (0.92–1.47)	1.45 (1.07–1.98)	1.19 (0.82–1.72)	1.33 (0.84–2.12)
Weighted mode	1.05 (0.89–1.24)	1.17 (0.95–1.44)	1.14 (0.88–1.49)	1.20 (0.88–1.63)
Simple mode	1.01 (0.78–1.29)	1.00 (0.68–1.46)	1.20 (0.79–1.81)	1.18 (0.70–1.98)
**HDL cholesterol (mg/dL)**				
Inverse variance weighted	1.29 (0.93–1.79)	1.17 (0.72–1.92)	0.91 (0.43–1.93)	1.39 (0.53–3.65)
Weighted median	1.26 (0.85–1.86)	1.00 (0.60–1.65)	1.02 (0.52–2.00)	1.01 (0.43–2.41)
MR‐Egger	1.96 (0.49–7.84)	0.66 (0.04–10.39)	6.26 (0.54–72.38)	26.75 (1.64–436.96)
Weighted mode	1.21 (0.74–1.98)	0.94 (0.52–1.70)	1.13 (0.49–2.60)	0.90 (0.36–2.25)
Simple mode	1.24 (0.80–1.91)	0.95 (0.49–1.85)	1.15 (0.49–2.69)	0.86 (0.29–2.56)
**LDL cholesterol (mg/dL)**				
Inverse variance weighted	1.04 (0.90–1.20)	1.14 (0.96–1.35)	1.09 (0.92–1.29)	1.07 (0.84–1.37)
Weighted median	1.05 (0.93–1.18)	1.16 (1.00–1.34)	1.09 (0.91–1.31)	1.09 (0.87–1.37)
MR‐Egger	1.07 (0.83–1.36)	1.21 (0.91–1.61)	1.10 (0.85–1.43)	1.23 (0.85–1.77)
Weighted mode	1.05 (0.93–1.18)	1.16 (0.99–1.36)	1.09 (0.90–1.33)	1.12 (0.87–1.44)
Simple mode	1.00 (0.76–1.32)	1.07 (0.74–1.57)	1.08 (0.75–1.55)	0.71 (0.38–1.33)
**Triglycerides (mg/dL)**				
Inverse variance weighted	0.73 (0.56–0.95)	0.79 (0.56–1.11)	0.73 (0.49–1.09)	0.69 (0.40–1.20)
Weighted median	0.71 (0.53–0.96)	0.81 (0.55–1.19)	0.73 (0.46–1.17)	0.89 (0.47–1.67)
MR‐Egger	0.60 (0.32–1.15)	0.58 (0.23–1.43)	0.88 (0.32–2.41)	0.60 (0.11–3.22)
Weighted mode	0.71 (0.48–1.04)	0.91 (0.51–1.62)	0.73 (0.40–1.35)	1.02 (0.39–2.65)
Simple mode	0.70 (0.47–1.05)	0.97 (0.52–1.81)	0.73 (0.40–1.32)	0.99 (0.35–2.80)

Abbreviations: CI, confidence interval; ER−, estrogen negative breast cancer; ER+ BC, estrogen positive breast cancer; OR, odds ratio; TNBC, triple‐negative breast cancer.

There was no evidence that TC was associated with the odds of overall BC (OR = 1.04, 95% CI = 0.93–1.18, *p* = 0.467) (Table S2). Similarly, we did not observe any causal relationship between HDL or LDL and the risk of BC (Table S2). The effect directions from the other five methods were consistent with IVW (Table S2). Estimates of all causal associations between lipids and overall BC are shown in Figure 2. In a reciprocal single‐trait MR test, we did not observe a relationship between the genetically determined risk of BC in each lipid trait (Table S12). The sensitivity analysis did not indicate significant heterogeneity between the SNPs, as shown by the Cochran *Q* test (Table 3). The MR‐Egger intercept *p* values were all above 0.05, showing no evidence of horizontal pleiotropy (Table 3). Additionally, our leave‐one‐out analysis revealed that no single SNP significantly influenced overall causal estimates (Figures S5–S8, Tables S5–S8). Scatterplots for MR analyses are presented in Figures S1–S4. Finally, the results of the MR‐PRESSO analysis did not show outliers (Table 3).

**TABLE 3:** MR results on heterogeneity and horizontal pleiotropy.

Exposure	Outcomes	Heterogeneity test						Pleiotropy test			MR‐PRESSO
		IVW			MR‐Egger			MR‐Egger intercept			Global test
		*Q*	*Q*_df	*Q*_*p*val	*Q*	*Q*_df	*Q*_*p*val	Intercept	SE	*p*	*p*
TC	Overall BC	8.793	9	0.457	7.762	8	7.762	−0.013	0.013	0.340	0.540
HDL	Overall BC	0.494	2	0.781	0.123	1	0.726	−0.028	0.046	0.652	—
LDL	Overall BC	6.803	4	0.147	6.664	3	0.083	−0.005	0.020	0.818	0.479
TG	Overall BC	0.542	3	0.910	0.138	2	0.933	0.019	0.029	0.590	0.935
TC	ER+ BC	12.452	9	0.189	8.616	8	0.376	−0.032	0.017	0.096	0.220
HDL	ER+ BC	2.959	2	0.228	2.517	1	0.113	0.039	0.092	0.747	0.220
LDL	ER+ BC	6.324	4	0.176	5.686	3	0.128	−0.013	0.023	0.602	0.364
TG	ER+ BC	3.281	3	0.350	2.572	2	0.276	0.031	0.041	0.535	0.439
TC	ER− BC	3.930	9	0.916	3.724	8	0.881	−0.009	0.020	0.662	0.924
HDL	ER−BC	4.455	2	0.108	1.264	1	0.261	−0.130	0.082	0.358	—
LDL	ER− BC	1.388	4	0.846	1.365	3	0.714	−0.003	0.021	0.889	0.919
TG	ER− BC	0.563	3	0.905	0.403	2	0.817	−0.018	0.046	0.728	0.918
TC	TNBC	3.568	3	0.312	3.514	2	0.173	−0.028	0.030	0.409	0.634
HDL	TNBC	4.553	2	0.103	0.011	1	0.917	−0.198	0.093	0.279	—
LDL	TNBC	4.957	4	0.292	3.798	3	0.284	−0.028	0.030	0.409	0.525
TG	TNBC	3.568	3	0.312	3.514	2	0.173	−0.018	0.046	0.728	0.349

Abbreviations: HDL, high‐density lipoprotein; LDL, low‐density lipoprotein; *p*, *p* value; *Q*, Cochran *Q* statistics; SE, standard error; TC, total cholesterol; TG, triglycerides.

### 3.2 MR With Outcome Stratified by ER Status

We performed MR analysis to test the relationship between genetically influenced lipids and BC risk stratified by ER+, ER−, and TNBC status. In these stratified analyses, we found no association between TC, HDL, LDL, and TG and all three subtypes of BC (Tables S3–S5). A heterogeneity test found no evidence to reject the null hypothesis of homogeneity between cancer subtypes (e.g., HDL: Cochran *Q* = 2.959, *p* = 0.228; Table 3). Therefore, we did not observe any substantive differences in the relationship of any lipid trait to ER+, ER−, or TNBC.

### 3.3 Multivariable MR

**FIGURE 4:**
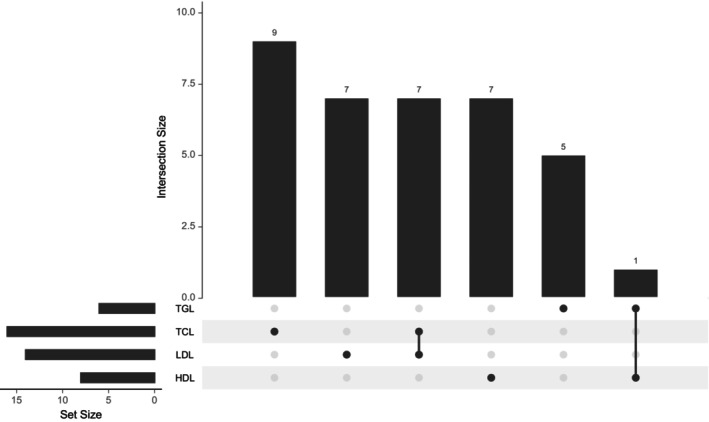
Overlap of instrumental variables between lipid traits. HDL, high‐density lipoprotein; LDL, low‐density lipoprotein; TC, total cholesterol; TG, triglycerides.

To test whether the association between lipid traits and BC risk remains robust when accounting for all related lipid factors, we conducted a MVMR analysis by simultaneously including all four lipid traits. Before further analysis, we compared the IVs used for the lipid traits and identified eight overlapping IVs. Of these, seven were shared between TC and LDL, whereas one IV overlapped between TG and HDL (Figure 4). We excluded overlapping SNPs, retaining 9, 7, 7, and 5 unique IVs for TC, LDL, HDL, and TG, respectively (Figure 4). These unique IVs were then used in the MVMR analysis to assess the independent effects of each lipid trait on the risk of BC. We did not observe significant relationships between genetically influenced HDL, LDL, TC, and TG with BC (HDL: OR = 0.113, 95% CI = 0.001–14.950, *p* = 0.415; LDL: OR = 3.048, 95% CI = 0.396–23.361, *p* = 3.048; TC: OR = 1.716, 95% CI = 0.416–7.059, *p* = 0.483; TG: OR = 6.359, 95% CI = 0.050–81.530, *p* = 0.398) (Table S10). Additionally, no association was detected when we considered the molecular subtypes (Table S10). A test for heterogeneity revealed no significant difference among the IVs (*p* > 0.05) (Table S11). Interestingly, our results were consistent with the univariable MR except for TG, whose negative causal association with overall BC was abrogated after accounting for the interrelationships between these lipid traits (Table S10).

## 4 Discussion

In this study, we investigated the causal relationship between genetically predicted blood lipid levels and BC risk among African women using MR. Our findings provide genetic evidence supporting an inverse causal relationship between genetically elevated TG and overall BC risk. In contrast, no significant causal associations were observed between genetically predicted TC, LDL, or HDL and overall BC risk. None of the serum lipids were found to be causally associated with the risk of ER+ BC, ER− BC, and TNBC. Furthermore, reverse causation analysis indicated no evidence that BC influences serum lipid levels. Sensitivity analysis confirmed that these results were not affected by horizontal pleiotropy.

Our findings align with previous studies [19, 45]. A recent MR study by Zhang et al. [46] also found that circulating TG levels were associated with a reduced risk of overall BC, HER2‐overexpressing, and luminal A BC but had no significant impact on other BC subtypes. This suggests that TG metabolism may play a role in the pathogenesis of BC, possibly across diverse genetic and environmental contexts. The protective effect of TGs in BC has been linked to their ability to shield cells from fatty acid‐induced damage [47]. A similar protective effect has been observed in other cancers, such as clear cell renal cell carcinoma (ccRCC) [48]. One possible explanation is that higher circulating TG levels act as an energy reservoir, reducing the availability of free fatty acids that contribute to oxidative stress and inflammation—two key drivers of breast carcinogenesis. However, some studies have reported conflicting findings. Research considering menopausal status has shown that higher TG levels are associated with an increased risk of BC [49, 50, 51]. Since BMI, weight, and menopausal status influence lipid metabolism and BC risk [52, 53, 54], their absence as covariates in our study may partly explain these discrepancies. Additionally, genetic and population‐specific variations in lipid metabolism could contribute to this inconsistency.

In the multivariate MR analysis, the association between TG and BC risk was attenuated after adjustment for TC, HDL, and LDL, indicating that TG levels do not affect BC risk independently. This finding is consistent with the study by Nowak and Ärnlöv [19] who reported an abolished association after excluding variants associated with other lipids. Although our analysis did not find a causal relationship between serum lipid levels and the risk of ER+ BC, ER− BC, and TNBC, it further emphasizes the complex involvement of lipids in the etiology of BC. The interplay between various lipid profiles, including TG, HDL, LDL, and TC, may contribute to the observed variability in lipid‐cancer associations across different populations and subtypes of BC.

The relationships between serum TC, LDL, and HDL with BC risk have been extensively explored, yet the results remain inconclusive. Some epidemiological studies have reported that elevated LDL is associated with BC risk because of its role in estrogen biosynthesis and oxidative stress [17]. Still, others have found no association or inverse relationship [13, 27]. Similarly, HDL has been proposed to have anti‐inflammatory and antioxidant properties that could theoretically protect against cancer development [18]. Some meta‐analyses and prospective studies have suggested a linear relationship [21, 45, 55], yet most studies also propose a protective role for HDL [56, 57]. Our MR analysis did not support a causal role for either TC, LDL, or HDL in BC risk among African women, suggesting that these lipid fractions may not be major independent drivers of carcinogenesis in this population. Our findings align with studies reporting no association [20, 58, 59]. Differences in genetic architecture, dietary patterns, and environmental exposures may contribute to the observed discrepancies across studies. Furthermore, estrogen metabolism plays a crucial role in hormone receptor‐positive BC, and cholesterol derivatives serve as precursors for estrogen synthesis. Future studies must incorporate hormonal status and menopausal transition to clarify whether the relationship between lipid profiles and BC varies by hormonal context.

Population‐specific analyses are crucial for understanding cancer risk factors in diverse genetic backgrounds. African populations exhibit distinct genetic variation patterns, including lipid‐related polymorphisms that may influence BC risk differently than European populations [60]. For instance, genetic variants influencing lipid metabolism in African populations may have different effect sizes or interact with unique environmental exposures, leading to population‐specific disease mechanisms. Our study contributes to the limited but growing body of literature on BC genetics in African women, highlighting the importance of conducting ancestry‐specific research to refine risk prediction models and intervention strategies.

Given the routine measurement of lipids in clinical settings, integrating lipid‐based risk models into BC screening strategies could improve early detection, particularly in resource‐limited settings. Additionally, our findings raise important questions about the potential impact of lipid‐modifying therapies, such as statins and TG‐lowering drugs, on BC risk. However, translating these findings into clinical applications is complex, as lipid metabolism is influenced by multiple factors, including BMI, menopausal status, and lifestyle behaviors [61, 62]. Future studies should incorporate these variables to refine risk prediction models and assess the potential utility of lipid‐targeted interventions in BC prevention.

### 4.1 Strengths and Limitations

This study possesses several significant strengths. First, it is the first study to use MR to determine the genetic association between lipids and BC in African women. We included the largest GWAS to date for BC among African women, consisting of 18,034 cases and 22,104 controls. The four lipid traits obtained from the AWI‐GEN study were associated with strong IVs, as indicated by *F*‐statistics well above the conventional threshold of 10, which satisfies the first assumption for MR analysis. Additionally, the population for both the exposure and outcome datasets consists entirely of African individuals, reducing potential biases related to population stratification. Using IVW, WM, MR‐Egger, and Cochran *Q* tests allowed us to control pleiotropic effects, a major concern in MR studies. The significant negative association between triglycerides and overall BC observed in the IVW model was consistent between the WM and MR‐Egger models and the MPRESSO algorithms. These findings suggest that elevated TG levels may potentially offer a protective effect against BC in African women.

As we have highlighted the strengths, there are also limitations to our study. The sample size, particularly for the lipid traits, was relatively small, which may have reduced the statistical power to detect associations. Moreover, due to the limited number of GWAS studies conducted among African populations, we were unable to account for potential confounders such as BMI, obesity, and other factors known to influence BC pathogenesis. Additionally, the study focused exclusively on data from individuals of African ancestry, which could limit the generalizability of the findings to other populations. Our findings highlight the need for expanded genomic studies in African populations. The limited availability of well‐powered GWAS datasets for lipid traits in non‐European populations may affect the precision of MR estimates. Given that most large‐scale genetic studies have been conducted in European cohorts, addressing these disparities is essential for improving the generalizability of genetic epidemiology findings. Increased investment in African genomic research will ensure that diverse populations benefit from advances in precision medicine.

## 5 Conclusion

The relationship between serum lipids and BC risk appears more complex than previously understood. To our knowledge, this study represents the first MR analysis investigating the causal relationship between serum lipid traits and the risk of BC, including the molecular subtypes in the African population. Our results suggest that genetically higher TG levels may have a protective effect against overall BC. This discovery provides insight into the etiological mechanisms through which TGs influence BC risk. In particular, it opens new avenues to explore the metabolic pathways that may underlie this association, potentially leading to the identification of novel biomarkers or therapeutic targets. Additionally, these findings have potential implications for public health and clinical strategies. However, further studies are warranted to elucidate the underlying biological mechanisms.

## Author Contributions

**Emmanuel Owusu Ansah:** conceptualization (lead), data curation (lead), formal analysis (equal), investigation (equal), methodology (equal), resources (equal), validation (equal), writing – original draft (equal), writing – review and editing (equal). **Foster Kyei:** conceptualization (equal), investigation (equal), resources (equal), writing – review and editing (equal). **Caleb Frimpong Opoku:** methodology (equal), validation (equal), writing – review and editing (equal). **Andrews Danquah:** project administration (equal). **Kwadwo Fosu:** methodology (equal), validation (equal). **Emmanuel Boateng Agyenim:** investigation (equal), methodology (equal). **Daniel Sakyi Agyirifo:** resources (equal), supervision (equal).

## Conflicts of Interest

The authors declare no conflicts of interest.

## Supporting information


Figures S1–S8

Tables S1–S12

## Data Availability

The datasets used and/or analyzed during the current study are presented in the manuscript. Summary statistics for GWAS are publicly available.
